# Cancer Immunotherapy: Theory and Application

**DOI:** 10.1155/2018/7502161

**Published:** 2018-06-21

**Authors:** Guobing Chen, Monica Bodogai, Norimasa Tamehiro, Chuanlai Shen, Jun Dou

**Affiliations:** ^1^Medical School, Jinan University, Guangzhou, Guangdong Province, China; ^2^National Institute on Aging, NIH, Baltimore, MD, USA; ^3^National Institute of Health Sciences, Tokyo, Japan; ^4^Southeast University Medical School, Nanjing, Jiangsu Province, China

In the last few decades, immunotherapy has become an important part of treating some types of cancer. Through either strengthening the host immune responses against tumors, supplying modified immune system components, or counteracting signals produced by cancer cells that suppress immune responses, immunotherapy has become an effective regimen alone or combined with other treatments, such as surgery, chemotherapy, and radiation therapy for cancer patients. With the rapid increase in our understanding of the immune system, more and more small molecules, peptides, recombinant antibodies, vaccines, and cellular therapeutic modalities are being applied to manipulate the immune response for cancer treatment. These immunotherapies have provided significant benefits against cancer, especially the application of immune checkpoint inhibitors [1] and cell therapies [2]. To reflect the advancement and diversity of this field, we invited prospective authors to contribute original manuscripts, case reports, clinical studies, and reviews that focused on antitumor immunotherapy.

In this issue, K. Łukasiewicz and M. Fol summarize the advantages and limitations of microorganisms for cancer treatment. Microorganisms, or a part of them, could stimulate the immune system generally or specifically to eliminate cancer cells. The microorganisms could also be developed as delivery vehicles with exceptional properties. However, the consideration of accompanied infection and limited types of the cancer candidates restricts the wide application of microorganisms, which need more attention and effort in the future.

R. Arai et al. observed decreased peripheral blood dendritic cell (DC) number and function with the lipid accumulation in lung cancer patients. DCs are critical antigen-presenting cells (APC) which present antigen peptides to T cells to initiate specific antitumor immune response. The accumulation of abnormal triglycerides in DC caused the decline of both APC number and function in cancer progression and metastasis. This offers a new potential antitumor target for research and development.

M. R. Rollins and R. M. Gibbons Johnson focused on PD-L1 in antitumor immunity. Checkpoint-associated antitumor therapy has recently had great successes in many types of cancers and advanced a new field that may have the potential to conquer some types of cancer. In this issue, Dr. Johnson's group demonstrated that activated CD8+ T cells could survive better without CD80 expression, which is one of the PD-L1 ligands. It raises the importance of CD80 in the design and implementation of checkpoint blockage for antitumor therapies.

Cell therapy is another excellent implementation with a rapid development in the last few years. Despite advanced manipulation, such as CAR-T and TCR-T therapies [3], the original tumor-infiltrating lymphocyte (TIL) therapy still demonstrated promising outcomes because of safety and longtime development. In a clinical study carried by W. Li et al., TIL combined with IFN-alpha therapy had significant, long disease-free survival and overall survival rates compared to that of no cell therapy in malignant melanoma patients.

Cereblon is a key protein in autosomal recessive nonsyndromic mental retardation and metabolic diseases because of the important regulation roles on the genes involved in cell proliferation and metabolism [4]. It also has different roles in immunomodulatory drug treatment of cancer patients. In this issue, Q. Shi and L. Chen summarize the function of cereblon in cell metabolism and generation of related diseases, as well as the multiple functions and mechanisms in the implementation of immunomodulatory drugs, which could greatly benefit any immunomodulatory drug.

Undoubtedly, there remain many more topics to be discussed, but this special issue includes a number of original research articles, clinical studies, and systematic reviews of cancer immunotherapy from different angles. We hope that this special issue can provide valuable information to researchers as well as clinicians and not only lead to enhancement of knowledge but also serve for better immunotherapy implementation for cancer patients.
